# Mountain spa rehabilitation improved health of patients with post-COVID-19 syndrome: pilot study

**DOI:** 10.1007/s11356-022-22949-2

**Published:** 2022-09-23

**Authors:** Anna Gvozdjáková, Zuzana Sumbalová, Jarmila Kucharská, Zuzana Rausová, Eleonóra Kovalčíková, Timea Takácsová, Plácido Navas, Guillermo López-Lluch, Viliam Mojto, Patrik Palacka

**Affiliations:** 1 Faculty of Medicine, Pharmacobiochemical Laboratory of 3rd Department of Internal Medicine, Comenius University in Bratislava, Sasinkova 4, 811 08 Bratislava, Slovakia; 2 Sanatorium of Dr. Guhr, 059 81 High Tatras, Tatranská, Polianka, Slovakia; 3 Centro Andaluz de Biología del Desarrollo, Universidad Pablo de Olavide-CSIC-JA, and CIBERER, Instituto de Salud Carlos III, Sevilla, Spain; 4 Faculty of Medicine and UNB, 3rd Department of Internal Medicine, Derer’s Hospital in Bratislava, Comenius University in Bratislava, Limbová 5, 833 05 Bratislava, Slovakia; 5 Faculty of Medicine, 2nd Department of Oncology, Comenius University in Bratislava, Klenová 1, 833 10 Bratislava, Slovakia

**Keywords:** High-altitude environment, Mountain spa rehabilitation, Post-COVID-19 syndrome, SARS-CoV-2, Pulmonary function, Clinical symptoms, Platelet mitochondrial metabolism, Coenzyme Q_10_, Oxidative stress

## Abstract

European Association of Spa Rehabilitation (ESPA) recommends spa rehabilitation for patients with post-COVID-19 syndrome. We tested the hypothesis that a high-altitude environment with clean air and targeted spa rehabilitation (MR — mountain spa rehabilitation) can contribute to the improving platelet mitochondrial bioenergetics, to accelerating patient health and to the reducing socioeconomic problems. Fifteen healthy volunteers and fourteen patients with post-COVID-19 syndrome were included in the study. All parameters were determined before MR (MR1) and 16–18 days after MR (MR2). Platelet mitochondrial respiration and OXPHOS were evaluated using high resolution respirometry method, coenzyme Q_10_ level was determined by HPLC, and concentration of thiobarbituric acid reactive substances (TBARS) as a parameter of lipid peroxidation was determined spectrophotometrically. This pilot study showed significant improvement of clinical symptoms, lungs function, and regeneration of reduced CI-linked platelet mitochondrial respiration after MR in patients with post-COVID-19 syndrome. High-altitude environment with spa rehabilitation can be recommended for the acceleration of recovery of patients with post-COVID-19 syndrome.

**Supplementary Information:**

The online version contains supplementary material available at 10.1007/s11356-022-22949-2.

## Introduction

The first new coronavirus originated from southeast China in 2003 (SARS—severe acute respiratory syndrome), and the second originated from Middle East in 2012 (MERS—Middle East respiratory syndrome) (Hilgefeld and Peiris 2013). In March 11, 2020, the World Health Organization (WHO) declared a global pandemic caused by the SARS-CoV-2 beta-coronavirus responsible for a new type of acute respiratory infection and an atypical pneumonia. WHO named the diseases caused by SARS-CoV-2 virus as “COVID-19” (Corona Virus Diseases 2019) (Wu et al. 2020). Persisting signs or symptoms related to SARS-CoV-2 infection can be divided into two categories. The first, subacute COVID-19 including symptoms present from 4 to 12 weeks beyond acute COVID-19 and second, post-COVID-19 syndrome (or chronic) including symptoms over 12 weeks after the SARS-CoV-2 infection (Fugazzaro et al. 2022). The main symptoms include shortness of breath, general fatigue, exhaustion, headaches, muscle and joint pain, cough, hair, taste and smell loss, sleep and memory disturbances, depression, sensitivity to sound and light, impaired quality of life and reduced daily activity (35%), reduced mobility (33%), and pain (33%) (Walle-Hansen et al. 2021). Taboada et al. (2021) reported limitations of everyday life near 50% of patients 6 months after hospitalization for COVID-19. In patients with severe SARS-CoV-2 infection, dyspnea develops that manifest as acute coronary distress syndrome (ACDS) and can lead to death (Wu et al. 2020).

SARS-CoV-2 viral infection occurs with higher incidence in patients with comorbidities such as diabetes mellitus type 2, obesity, cardiovascular disease, chronic lung disease, and cancer (Zhang and Liu 2020; Shi et al. 2018; Huang et al. 2020; Li et al. 2020). In aged people, dysfunctions of immune system and mitochondrial health are key factors in COVID-19 disease (Lopez-Lluch 2017; Fernandez-Ayala et al. 2020; Ganji and Reddy 2021). Mechanical ventilation is required primarily in patients with comorbidities (Siddiq et al. 2020). In patients with post-COVID-19 syndrome, individualized rehabilitation programs are recommended, focused to pulmonary rehabilitation of individuals with post-COVID-19 syndrome (NICE 2022). ESPA, Wang et al. (2020), and Maccarone and Mesiero (2021) recommend spa pulmonary rehabilitation for patients with post-COVID-19 syndrome.

Virus proteins need mitochondria for their survival and replication. Mitochondria play the central role in the primary host defense mechanisms against viral infections (Gvozdjáková et al. 2020). Many viruses modulate mitochondrial function, producing more reactive oxygen species, (ROS), cytokine storm, and stimulate inflammation (Ganji and Reddy 2021; Gordon et al. 2020). SARS-CoV-2 infection caused oxidative stress, mitochondrial dysfunction, platelet dysfunction and coagulation (Ohta and Nishiyama 2011; Archer et al. 2020), and high morbidity and mortality. SARS-CoV-2 virus may manipulate mitochondrial dynamics, metabolism, mitochondrial bioenergetics, apoptosis and antiviral immunity and alter intracellular distribution of mitochondria.

**Fig. 1:**
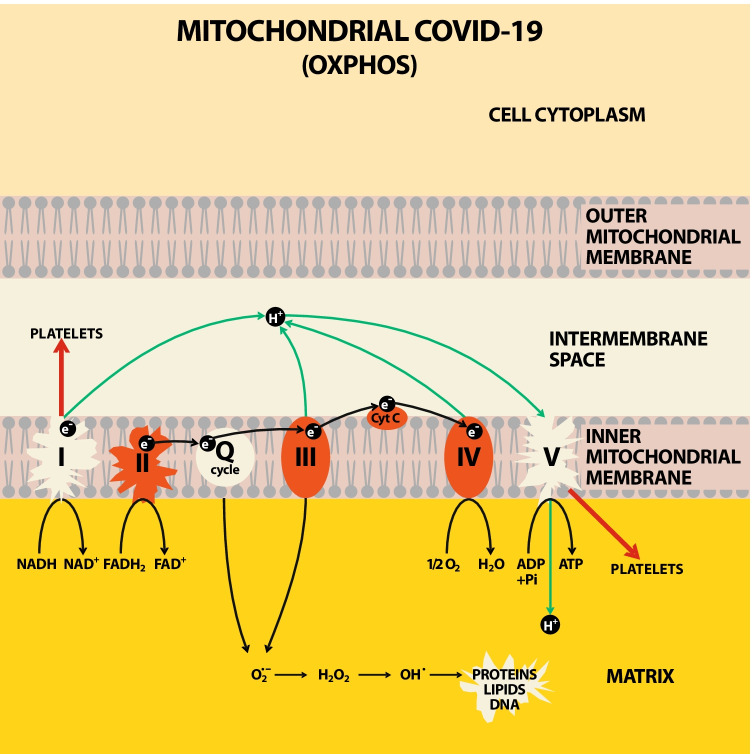
Effect of SARS-CoV-2 on platelet mitochondrial respiratory chain and oxidative phosphorylation in patients after acute COVID-19. Legend: SARS-CoV-2 in platelet mitochondria of patients after overcoming the disease COVID-19 decreased the function of mitochondrial respiratory chain at complex I, endogenous level of coenzyme Q_10_ in Q-CYCLE, ATP production by oxidative phosphorylation — Complex V.; respiratory chain complexes: I, II, III, IV, V; Q-cycle of coenzyme Q_10_; cyt c — cytochrome c; e^−^ — electron; NADH — reduced nicotinamide adenine dinucleotide; NAD^+^ — nicotinamide adenine nucleotide; FADH_2_ — flavin adenine dinucleotide reduced; FAD^+^ — flavin adenine nucleotide; O_2_^−^ — superoxide radical; H_2_O_2_ — hydrogen peroxide; proteins; lipids, DNA — deoxyribonucleic acid; O_2_ — oxygen; H_2_O — water; ADP — adenosine diphosphate; ATP — adenosine triphosphate; Pi — inorganic phosphate

In 2020, we published the hypothesis that mitochondrial bioenergetics and endogenous coenzyme Q_10_ (CoQ_10_) level could be targets of the new SARS-CoV-2 virus (Gvozdjáková et al. 2020). Currently, this hypothesis was proved by authors who showed reduced mitochondrial bioenergetics in monocytes (Gibellini et al. 2020) and in peripheral blood mononuclear cells of patients with COVID-19 (Ajaz et al. 2021). Our pilot study show reduced platelet mitochondrial function with deficit of endogenous CoQ_10_ level in non-hospitalized, non-vaccinated patients 3–6 weeks after acute COVID-19 (Sumbalová et al. 2022). The effect of SARS-CoV-2 virus on mitochondrial respiratory chain was named “Mitochondrial COVID-19” (Gvozdjáková et al. 2022) (Fig. 1).

New strategies for COVID-19 prevention and therapy are being sought to reduce the negative effects of SARS-CoV-2 virus in society. Environmental strategies play a vital role in pandemic prevention similar to COVID-19. Reduction of air quality can support the transmission dynamics of infectious disease in society with consequential socioeconomic problems (Coccia 2021; Coccia 2021b; Coccia 2022). To the best of our knowledge, the effect of SARS-CoV-2 and high-altitude environment with targeted spa rehabilitation on pulmonary function, platelet mitochondrial bioenergetics, coenzyme Q_10_ level (CoQ_10_), (a key mitochondrial component for energy production), and lipid peroxidation of patients with post-COVID-19 syndrome has not been described. MR is beneficial for chronic pulmonary diseases, improving fatigue, joint pain, psychological stress, sleep disorders, and quality of life in patients with various diseases (Gvozdjáková et al. 2021).

We tested other hypothesis and strategy for patients with post-COVID-19 syndrome that a high-altitude environment with clean air and targeted spa rehabilitation of patients with post-C-19 syndrome can contribute to improving platelet mitochondrial bioenergetics, to accelerating patients’ health and to the reducing socioeconomic problems.

## Materials and methods

### Subjects

#### The control group (C)

The control group (C) consisted of fifteen healthy individuals (6 men and 9 women), aged 38 to 67 years with a mean age of 51.3 ± 2.3 years, BMI 25.2 ± 0.9 kg/m^2^. The inclusion criteria for healthy subjects were absence of chronic medication and no history of COVID-19. Exclusion criteria were lung and heart diseases, diabetes, cancer, obesity, smoking, and regular alcohol consumption.

#### The group of patients with post-COVID-19 syndrome (MR)

In May and June of 2021, fourteen patients with post-COVID-19 syndrome, from Sanatorium of Dr. Guhr, High Tatras, Tatranská Polianka in Slovakia, were included in this study (MR group—mountain spa rehabilitation). Ten of them returned questionnaire of clinical symptoms before and after MR. The group of patients at the time of admission to mountain spa rehabilitation are marked as MR1 group, the same group of patients after mountain spa rehabilitation is marked as MR2 group. The mean age of the patients was 58.69 ± 2.64 years, (8 men and 6 women), BMI 29.85 ± 1.54 kg/m^2^.

#### COVID-19 history of the patients with post-COVID-19 syndrome

The patients were hospitalized for three weeks in the period from November 2020 to April 2021 for COVID-19. The causes for hospitalization of these COVID-19 patients were increased body temperature between 37.5 and 39.4°C (*n* = 8), bilateral pneumonia (*n* = 9), asthma bronchiale (*n* = 2), dyslipoproteinemia (*n* = 8), and the necessity of oxygen therapy (*n* = 8). In the patients, many clinical and psychological symptoms persisted during next 3–6 months after hospitalization classified as post-COVID-19 syndrome. The main symptoms on admission to MR were fatigue, cough, loss of smell, impaired breathing during exercise, loss of hair, and depression. In some patients, the loss of appetite was accompanied with considerable weight loss.

### Functional capacity of the lungs

The functional capacity of the lungs was evaluated in ten of the fourteen patients by 6-min walking test (6MWT) (Brooks et al. 2002; Casanova et al. 2011), exercise dyspnea during 6MWT by Borg scale (BS) (Borg 1982), and blood oxygen saturation (SpO_2_) before and after 6MWT. The results are summarized in Table 1. These tests were performed before (MR1) and after mountain spa rehabilitation (MR2). Blood samples were collected the first morning after admission to the mountain spa before (MR1) and after 16–18 days of mountain spa rehabilitation (MR2).

**Table 1:** Effect of MR on lungs function of patients with post-COVID-19 syndrome

Parameter	MR1 (*n* = 10)	MR2 (*n* = 10)	MR2 vs MR1 *p* value
6MWT (m)	479 ± 40.9	566.2 ± 23.3	0.018^x^
BS (number)	5.9 ± 0.8	3.8 ± 0.5	0.004^xx^
SpO_2_ **(%)**			
Before 6MWT	94.1 ± 0.59	94.1 ± 0.72	ns
After 6MWT	94.9 ± 0.60	93.9 ± 0.78	ns

*6MWT* 6-min walking text; *BS* Borg scale; *SpO*_*2*_ blood oxygen saturation; *MR1* the patients with post-COVID-19 syndrome at the beginning of the study; *MR2* the patients with post-COVID-19 syndrome after 16–18 days of MR; ^x^*p*<0.05, ^xx^*p*<0.01 vs MR1

### Clinical symptoms of patients with post-COVID-19 syndrome

Patients completed a questionnaire (21 questions) before and after MR. The results are summarized in Table 2.

**Table 2:** Effect of MR on clinical symptoms of patients with post-COVID-19 syndrome

Clinical symptom	Before MR (MR1) (number of symptoms)	After MR (MR2) (number of symptoms)
Dry cough	3	3
Difficulty breathing	6	3
Shortness of breath in rest	4	3
Elevated temperature	2	0
Chills	2	1
Heart palpitations	3	1
Respiratory support with oxygen	0	0
Weakness	0	0
Overall fatigue	7	2
Malaise	2	2
GIT problems	0	0
Diarrhea	1	1
Chest pain	3	1
Muscle and joint pain	10	5
Back pain	0	0
Headache	4	0
Loss of taste and smell	0	0
Weight loss	1	1
Hearing impairment	2	0
Visual disturbance	3	1

### Blood count and biochemical parameters

In all patients with post-COVID-19 syndrome blood counts, blood lipid parameters, glucose, and CRP were determined in Hospital of Dr. Vojtech Alexander in Kežmarok, High Tatras, Slovakia. The determined parameters are summarized in Table 3.

**Table 3:** Effect of MR on blood count and metabolites of patients with post-COVID-19 syndrome

	Control (*n* = 15)	MRl (*n* = 14)	MR2 (*n* = 14)	MR1 vs C *p* value	MR2 vs MR1 *p* value
Blood count					
WBC (10^9^/L)	6.23 ± 0.47	6.99 ± 0.72	6.59 ± 0.64	0.396	0.327
RBC (10^9^/L)	4.66 ± 0.12	4.62 ± 0.12	4.80 ± 0.09	0.717	0.008 ^xx^
HCT (ratio)	0.410 ± 0.100	0.418 ± 0.01	0.438 ± 0.008	0.813	0.003 ^xx^
PLT (10^9^/L)	247.5 ± 16.1	213.9 ± 14.9	219.1 ± 11.2	0.154	0.556
MCV (fL)	87.14 ± 0.65	90.31 ± 1.26	91.21 ± 1.26	0.024*	0.009 ^xx^
MCH (pg)	29.95 ± 0.28	31.58 ± 0.49	31.10 ± 0.41	0.014*	0.079
MCHC (g/L)	343.71 ± 2.53	349.61 ± 2.00	341.08 ± 1.21	0.273	0.002 ^xx^
HgB (g/L)	140.67 ± 3.32	145.46 ± 3.44	149.23 ± 2.89	0.520	0.056
Lipid parameters					
CHOL (mmol/L)	5.32 ± 0.27	5.507 ± 0.299	5.76 ± 0.397	0.707	0.264
HDL-CH (mmol/L)	1.41 ± 0.13	1.100 ± 0.086	1.121 ± 0.099	0.031*	0.632
LDL-CH (mmol/L)	3.09 ± 0.25	3.368 ± 0.287	3.344 ± 0.316	0.319	0.904
TAG (mmol/L)	2.05 ± 0.49	2.489 ± 0.555	3.224 ± 0.954	0.055	0.142
Other parameters					
CRP (mg/L)	0.90 ± 0.20	1.80 **±** 0.45	1.81 ± 0.53	0.721	0.950
GLU (mmol/L)	5.13 ± 0.17	6.17 ± 0.63	5.20 ± 0.26	0.139	0.069

*MR1* The patients before mountain spa rehabilitation; *MR2* the patients after mountain spa rehabilitation; *WBC* white blood cells, *RBC* red blood cells, *HCT* hematocrit, *PLT* platelets, *MVC* mean corpuscular volume, *MCH* mean corpuscular hemoglobin, *MCHC* mean corpuscular hemoglobin concentration, *HgB* hemoglobin, *CHOL* total cholesterol, *HDL-CH* HDL cholesterol, *LDL-CH* LDL cholesterol, *TAG* triacylglycerols, *CRP* c-reactive protein, *GLU* glucose. Data are presented as mean ± sem. The differences between MR1 and the control group, and between MR2 and MR1 group are statistically evaluated, *p<0.05 vs control, ^XX^*p*<0.01 vs MR1

### Coenzyme Q_10_ determination

Total coenzyme Q_10_ concentration (ubiquinol + ubiquinone) in whole blood, plasma, and isolated platelets were estimated using an isocratic HPLC method (Lang et al. 1986; Kucharská et al. 1998). For the oxidation of ubiquinol to ubiquinone, 1,4-benzoquinone was added to blood or plasma sample (Mosca et al. 2002). The concentrations of CoQ_10-TOTAL_ were calculated in μmol/L. The isolated platelets were disintegrated with methanol (Niklowitz et al. 2004). Concentrations of CoQ_10-TOTAL_ in platelets were calculated in pmol/10^9^ cells.

### TBARS

A parameter of oxidative stress — an indicator of lipid peroxidation in plasma — was determined spectrophotometrically by measuring the formation of thiobarbituric acid reactive substances (TBARS) (Janero and Bughardt 1989). The concentration in μmol/L was calculated.

### Platelets preparation

Platelets were isolated from whole blood as described previously (Sumbalova et al. 2018; Palacka et al. 2022) and counted on hematological analyzer Mindray BC-3600 (Mindray, China).

### High-resolution respirometry

**Fig. 2:**
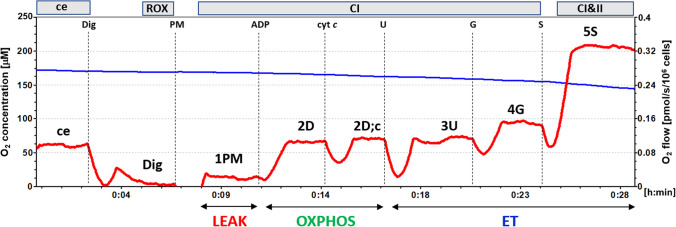
The trace from the measurement of platelet mitochondrial respiration in freshly isolated platelets (Doerrier et al. 2016). Legend: The blue line shows oxygen concentration (μM) and the red trace oxygen consumption (pmol O2/s/10^6^ cells). 250 × 10^6^ platelets were added into a 2-mL chamber of an O2k-Respirometer with mitochondrial respiration medium MiR05 plus 20 mM creatine at 37 °C and continuous stirring (750 rpm). The titration steps are cells (ce), digitonin (Dig); pyruvate plus malate (PM); adenosine diphosphate (ADP); cytochrome c (cyt c); uncoupler FCCP (U); glutamate (G); and succinate (S). All substrates were added in kinetically saturating concentrations; FCCP was titrated in optimum concentration to reach the maximum O_2_ flow. ce — intact cells; ROX — residual oxygen consumption; CI — complex I pathway; CI&II — complex I and complex II pathway; LEAK — non-phosphorylating resting state of respiration (*L*); OXPHOS — the phosphorylating state of respiration (*P*); ET — noncoupled state of respiration at optimum concentration of uncoupler

The mitochondrial bioenergetics in platelets was evaluated by high-resolution respirometry (HRR) method (Pesta and Gnaiger 2012; Sjovall et al. 2013). For the respirometric assay, 250×10^6^ platelets were used in a 2-mL chamber of an O2k-Respirometer (Oroboros Instruments, Austria). The respiration was measured at 37°C in mitochondrial respiration medium MiR05 with addition of 20 mM creatine, and under continuous stirring at 750 rpm. The data were collected with DataLab software (Oroboros Instrument, Austria) using a data recording interval of 2s (Pesta and Gnaiger 2012; Doerrier et al. 2016). For evaluation of platelet mitochondrial bioenergetics, substrate-uncoupler-inhibitor (SUIT) protocol 1 (Doerrier et al. 2016) was applied (Gvozdjáková et al. 2019). The representative trace is in Fig. 2.

The cell suspension volume containing 250 × 10^6^ platelets was added to the 2-mL chamber of O2k-Respirometer filled with the respiration medium. After stabilization at ROUTINE respiration of intact platelets, digitonin (0.20 μg/10^6^ cells) was added for plasma membrane permeabilization. Next, the chemicals were added in following order: 1PM–CI-linked substrates pyruvate (5 mM) and malate (2mM) were added to fuel CI-linked LEAK respiration; 2D — saturating ADP (1 mM) was added for determination of CI-linked respiratory capacity of oxidative phosphorylation (OXPHOS); 2D; c — cytochrome c (10 μM) was added for testing the outer mitochondrial membrane integrity; 3U — uncoupler FCCP (0.5 μM) was added at optimum concentration for determination of electron transfer (ET) capacity with CI-linked substrates pyruvate+malate; 4G — glutamate (10 mM) was added for evaluation of ET capacity with CI-linked substrates pyruvate+malate+glutamate; 5S — CII-linked substrate succinate (10 mM) was added for determination of CI&CII-linked ET capacity. For evaluation of mitochondrial pathway–related rates (here labeled according to titration steps), the rate after digitonin representing residual oxygen consumption (ROX) was subtracted from all respiratory rates (Fig. 2).

### Citrate synthase

The activity of citrate synthase as mitochondrial marker was evaluated spectrophotometrically according to the method of Srere (1969a, 1969b), described in detail by Eigentler et al. (2020). The activity of CS is evaluated in μmol/min/10^6^ cells.

### Data analysis

The differences between parameters of the post-COVID-19 MR1 group and the control group were evaluated using unpaired Student’s *t* test. For evaluation, the difference between MR1 and MR2 paired Student’s *t* test was used. *P* values <0.05 were considered statistically significant. The results are shown as individual points and the mean ± standard error of mean (sem).

## Results

Pulmonary function of patients with post-COVID-19 syndrome was evaluated by 6-min walking test (6MWT), exercise dyspnea by Borg scale (BS), and blood oxygen saturation (SpO2). By 6MWT, the distance that a patient can quickly walk in a period of 6 min is measured, reflecting the functional pulmonary capacity. In our patients, 6MWT test improved significantly after MR (from 479 ± 40.9 m to 566.2 ± 23.3 m, *p* = 0.018), the walked distance during the 6MWT increased by 87.2 m. Exercise dyspnea was measured by BS points from 0 to 10. Zero on BS means no dyspnea and 10 points on BS reflect maximal dyspnea after 6MWT. Exercise dyspnea measured by BS statistically significantly improved in patients with post-COVID-19 syndrome after MR by 2.1 points (from 5.9 ± 0.8 points to 3.8 ± 0.5 points, *p* = 0.004). Physiological levels of SpO_2_ are between 95 and 100%. SpO_2_ before 6MWT and after 6MWT were without significant changes after MR (Table 1).

### Effect of MR on clinical symptoms of patients with post-COVID-19 syndrome

From fourteen patients, ten patients filled out the questionnaire for evaluation of clinical symptoms before and after MR. Several patients had more than three clinical symptoms of COVID-19 before MR. Many clinical symptoms have improved after MR, as breathing difficulty, shortness of breath, chills, heart palpitations, overall fatigue, muscle and joint pain, chest pain, headache, hearing impairment, and visual disturbance (Table 2).

### Effect of MR on blood count and metabolites of patients with post-COVID-19 syndrome

MR significantly improved blood count, as the count of RBC (*p* = 0.008), HCT (*p* = 0.003), MCV (*p* = 0.009), and HgB (*p* = 0.056) were higher in MR2, and MCHC was lower (*p* = 0.002) compared to MR1. Mean of lipids parameters (CHOL, HDL-CH, LDL-CH, TAG) of post-COVID-19 patients showed dyslipoproteinemia. These parameters were not influenced by mountain spa rehabilitation (Table 3). CRP was higher in patients with post-COVID-19 syndrome vs control and did not improve after MR. Slightly higher blood glucose level of the patients improved after MR (*p* = 0.069, Table 3).

### Effect of MR on impaired platelet mitochondrial bioenergetics in patients with post-COVID-19 syndrome

**Fig. 3:**
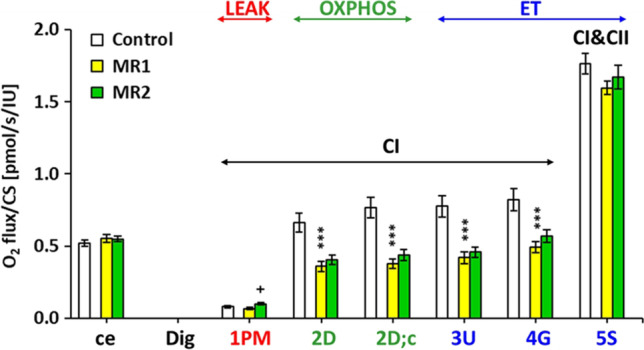
Effect of mountain with spa rehabilitation on platelet mitochondrial bioenergetics in patients with post-COVID-19 syndrome. Legend: ce: ROUTINE respiration of intact platelets; 1PM: complex I-linked LEAK (state 4) respiration with substrates (pyruvate + malate); 2D: complex I-linked OXPHOS (state 3) respiration capacity associated with CI-linked ATP production; 2D;c: The OXPHOS capacity after cytochrome c addition; 3U: The respiration after uncoupler FCCP titration represents CI-linked electron transfer (ET) capacity with substrates pyruvate+malate; 4G: ET capacity with substrates pyruvate+malate+glutamate; 5S: CI&CII-linked ET capacity with substrates pyruvate + malate + glutamate + succinate, (Doerrier et al. 2016; Gvozdjáková et al. 2019). The respiratory rates are marked according the steps in the SUIT protocol 1 (see Fig. 2). Control — the control group; MR1 — patients before mountain spa rehabilitation; MR2 — patients after mountain spa rehabilitation. CI — complex I pathway; CI&CII — complex I and complex II pathway; LEAK — the non-phosphorylating resting state of respiration; OXPHOS — the phosphorylating state of respiration; ET — the noncoupled state of respiration at optimum uncoupler concentration

We used freshly isolated platelets from patients with post-COVID-19 syndrome before MR (MR1) and after 16–18 days of special MR (MR2). All platelet respiratory parameters are expressed as *JO*_*2*_*/CS* (pmol/s/IU). The results of platelet mitochondrial bioenergetics analysis are shown in Fig. 3 and in supplementary material Fig. S3A-S3H.

Detailed results are shown in supplementary material, Fig. S3A–S3H. Routine respiration of intact platelets (ce) was similar in all groups (Fig. 3, Fig. S3A). The rate of mitochondrial LEAK respiration with CI-linked substrates (1PM) in MR1 group was lower (by 14.2%), although not statistically significant in comparison with control group. In MR2 group, mitochondrial LEAK respiration with CI-linked substrates was significantly increased vs MR1 (by 47.8%, *p* = 0.029, Fig. 3; Fig. S3B). CI-linked respiration coupled with ATP production (2D — CI-linked oxidative phosphorylation (OXPHOS) capacity) in the MR1 group was significantly reduced (*p* = 0.0004) by 45.8% vs control group values. In MR2 group, this parameter associated with ATP production was slightly improved (by 12.3% vs MR1) (Fig. 3; Fig. S3C). The respiration after addition of cytochrome c (2D;c) in the MR1 group was decreased by 50.6% vs control group values (*p* = 0.00002), in MR2 group, this parameter was slightly improved vs MR1 group (by 15.3%) (Fig. 3; Fig. S3D). Maximal mitochondrial oxidative capacity (the electron transfer capacity, ET) after uncoupler titration (3U) was significantly reduced in MR1 group vs control group (by 45.7%, *p* = 0.0002). In MR2 group, this parameter was slightly improved vs MR1 (by 8.8%) (Fig. 3; Fig. S3E). After addition of CI-linked substrate glutamate (4G), the ET capacity was significantly lower in MR1 group vs control group (by 40.0%, *p* = 0.0005). This respiration was slightly increased in MR2 group vs MR1 (by 15.6%, Fig. 3; Fig. S3F). ET capacity with CI&II-linked substrates (5S) was lower in MR1 group vs control group (by 9.7%, *p* = 0.060). This parameter was slightly higher in MR2 vs MR1 group (by 4.8%, Fig. 3; Fig. S3G). The mean improvement of mitochondrial parameters representing OXPHOS and ET capacity was 11.4% (from 4.8 to 15.6%) in comparison with MR1 group, which was taken as 100% (Fig. 3; Fig. S3C – S3G).

**Fig. 4:**
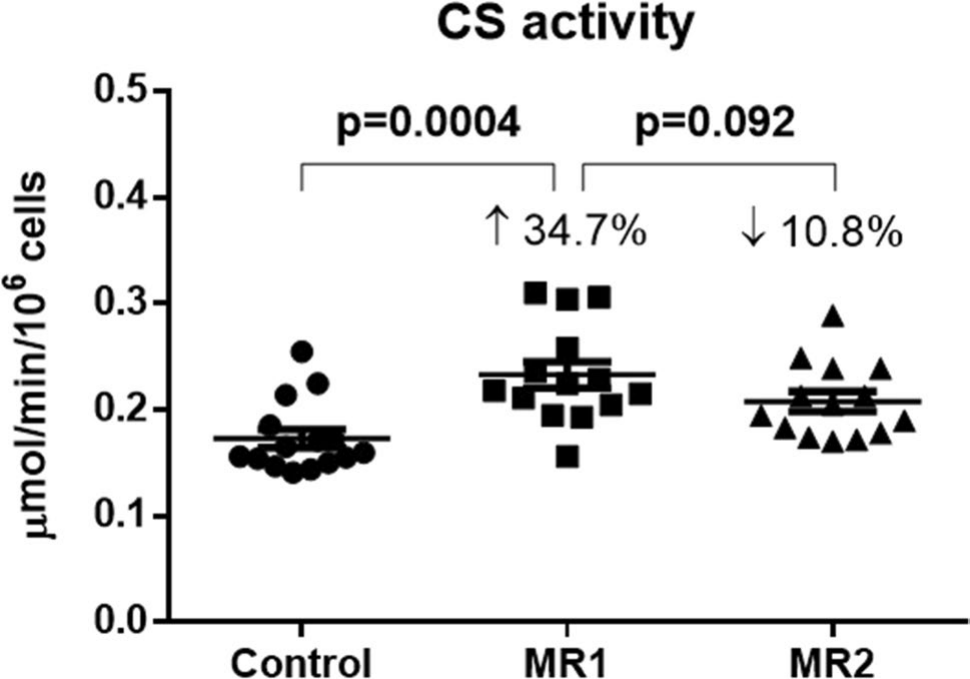
Effect of MR on citrate synthase activity in platelets of patients with post-COVID-19 syndrome. Legend: CS — citrate synthase; MR1 — before mountain spa rehabilitation; MR2 — after mountain spa rehabilitation

The mitochondrial marker — the activity of citrate synthase — was increased in patients with post-COVID-19 syndrome in comparison with control group (by 34.7%, *p* = 0.0004). After MR, the activity of citrate synthase in platelets slightly decreased vs MR1 (by 10.8%, *p* = 0.092, Fig. 4).

### Effect of MR on TBARS and endogenous CoQ_10_ in patients with post-COVID-19 syndrome

There was no significant difference in TBARS concentration between control group and patients with post-COVID-19 syndrome. Endogenous concentration of CoQ_10-TOTAL_ (ubiquinone + ubiquinol) in platelets, blood, and plasma of the post-COVID-19 syndrome group did not significantly differ from the control group and did not change after MR (Table 4).

**Table 4:** Effect of MR on lipid peroxidation and CoQ_10-TOTAL_ concentration of patients with post-COVID-19 syndrome

Parameter	Control (*n* = 15)	MR1 (*n* = 14)	MR2 (*n* = 14)
TBARS in plasma (μmol/L)	4.80 ± 0.18	4.65 ± 0.16	4.52 ± 0.17
CoQ_10-TOTAL_ in:			
Platelets (pmol/10^9^ cells)	84.14 ± 5.56	93.92 ± 5.92	91.47 ± 7.11
Blood (μmol/L)	0.313 ± 0.020	0.366 ± 0.035	0.315 ± 0.017
Plasma (μmol/L)	0.516 ± 0.032	0.516 ± 0.045	0.509 ± 0.035

*TBARS* indicator of lipid peroxidation; *CoQ*_*10-TOTAL*_ ubiquinol + ubiquinone; *MR1* before mountain spa rehabilitation; *MR2* after mountain spa rehabilitation;

## Discussion

WHO recommended rehabilitation (WHO 2022) and ESPA recommended spa rehabilitation of the patients with post-COVID-19 syndrome. The rehabilitation improved pulmonary function, exercise capacity, and quality of life of patients with post-acute phase of COVID-19 (Nalbandian et al. 2021; Schaeffer et al. 2022). Other authors used rehabilitation based on respiratory physiotherapy techniques, on exercise training or in combination with yoga (Srinivasan et al. 2021; Herrera et al. 2021).

The current pilot study was undertaken to determine the effect of high-altitude environment and targeted rehabilitation in spa on pulmonary function, clinical symptoms, endogenous coenzyme Q_10_ levels, oxidative stress, and platelet mitochondrial oxidative phosphorylation (OXPHOS) function in patients with post-COVID-19 syndrome. In high-altitude environment of High Tatras in Slovakia, spa rehabilitation in Sanatorium of Dr. Guhr, Tatranská Polianka is used for curing chronic pulmonary diseases for many years. The Sanatorium is located at altitude of 1005 m above sea level, in the zone of forests with dry air, favorable solar radiation, reduced partial oxygen pressure and air pressure, and with a mild, relatively stable daily temperature (Gvozdjáková et al. 2019). For patients with post-COVID-19 syndrome in high-altitude environment and spa rehabilitation program include walking, breathing exercises, oxygen therapy, exercise, water procedures, massages, psychological support, and education (Jendrichovsky et al. 2021; Tiku et al. 2020). The rehabilitation program is individualized for the improvement of mental health, to prevent skeletal muscle hypotrophy with a focus on increasing the rate of daily movement and overall patient activity.

Beneficial effect of pulmonary rehabilitation was documented in patients with chronic respiratory disease (Spruit et al. 2013). Improvements in exercise capacity, dyspnea, fatigue, anxiety, and depression after a pulmonary rehabilitation were reported by Soril et al. (2022). We evaluated pulmonary function of patients with post-COVID-19 syndrome by 6MWT, BS, and SpO_2_. By 6-min walking test, the distance that a patient can quickly walk in a period of 6 min (6MWT) is measured, reflecting the functional pulmonary capacity (Brooks et al. 2002; Casanova et al. 2011). After MR2 6MWT improved significantly (*p* = 0.018), the walking distance increased by 87.2 m. The increase in 6MWT by 70 m is considered clinically important for patients. Exercise dyspnea was evaluated by Borg scale (Borg 1982). After MR2, the exercise dyspnea was significantly improved (*p* = 0.004). An improvement by 0.5 point on BS is considered as an improvement of lung function. Oxygen saturation (SpO_2_) levels before and after 6MWT were without significant changes after MR (Table 1).

Rehabilitation of patients in a high-altitude environment reduced the extent of physical, cognitive, and mental impairment (as breathing, total fatigue, muscle, joint and chest pain, headache, memory impairment, depression, hearing impairment, visual disturbances), and improved the quality of life of patients with post-COVID-19 syndrome (Table 2). Although special rehabilitation in the Sanatorium lasted only 16–18 days, the positive effect of MR was manifested in patients with post-COVID-19 syndrome.

Several pathobiochemical mechanisms participate in virus infection on cellular and subcellular level. Mitochondria (subcellular particles) play a central role in the primary host defense mechanisms against viral infections, and in these processes, a number of novel viral and mitochondrial proteins are involved. One possible mechanism of SARS-CoV-2 effect is a manipulation of mitochondrial bioenergetics indirectly, by ACE2 regulation, and the other possibility is manipulation by localizing ORF-9b (open reading frame) protein to mitochondria. Manipulations of host mitochondria by viral ORFs can release mtDNA in the cytoplasm, activate mtDNA-induced inflammasome, and suppress innate and adaptive immunity (Singh et al. 2020; Singh et al. 2021). In the pathological conditions, as by virus activated cells, their request for energy production is increased (Singh et al. 2020). Under physiological conditions, platelets receive approximately 60% of energy from glycolysis and 40% energy from OXPHOS (Gatti et al. 2020; Warburg et al. 1927). Other mechanism of SARS-CoV-2 virus is its role in manipulating mitochondrial function. SARS-CoV-2 hijacks of host mitochondria of immune cells in COVID-19 disease (Singh et al. 2020), and impairs mitochondrial dynamics leading to cell death (Ganji and Reddy 2021; Seth et al. 2005). Mitochondrial “hijacking” by SARS-CoV-2 virus could be a key factor in the pathogenesis of this virus and induction of COVID-19 (Saleh et al. 2020; Singh et al. 2021). A good mitochondrial fitness could be considered as a protective factor against viral infections, including COVID-19 (Maccarone and Mesiero 2021; Burtscher et al. 2021; Jimeno-Almazán et al. 2021).

Mitochondrial antiviral signaling protein (MAVS), associated with the outer mitochondrial membrane, mediates the activation of NF_*K*_-B and the induction of interferons in response to viral infection (Sun et al. 2006; Seth et al. 2005). Many viruses target mitochondrial metabolism, dynamics, mitochondrial bioenergetics, membrane potential, ion permeability, induce reactive oxygen species production, alter the Ca^2+^ regulatory activity, and cause oxidative stress in host cells (Anand and Tikoo 2013; Elesela and Lukacs 2021). Viruses can modulate apoptosis and mitochondrial antiviral immunity, alter intracellular distribution of mitochondria, cause host mitochondrial DNA depletion for their survival in the cell (Ohta and Nishiyama 2011; Ripoli et al. 2010; Di Gennaro et al. 2020). Progression of the disease in COVID-19 patients involves “cytokine storm” with iron dysregulation (as hyperferritinemia) which induces ROS production and oxidative stress (Saleh et al. 2020).

The regeneration of mitochondria impaired by SARS-CoV-2 viruses can be achieved by various means including breathing exercises, increased physical activities, reduction of daily calories intake, enhanced daily intake of food with antioxidants properties (Ganji and Reddy 2021), spa rehabilitation (Wang et al. 2020; Maccarone and Mesiero 2021), and targeted coenzyme Q_10_ supplementation (Gvozdjáková et al. 2019). This pilot study showed significant deficit of platelet mitochondrial complex I-linked ET capacity and OXPHOS respiration associated with ATP production in patients with post-COVID-19 syndrome which were improved by spa rehabilitation in a high-altitude environment.

An essential component of the mitochondrial respiratory chain for energy (adenosine triphosphate) production is coenzyme Q_10_ (CoQ_10_) with antioxidant properties. In physiological conditions, CoQ_10_ transports electrons from complex I and complex II to complex III. Complexes of respiratory chain (CI, CIII, and IV) are organized in supercomplexes minimizing the distance for electron transfer. In the pathological conditions, electron flux from CoQ can be reversed to CI reducing NAD^+^ — the phenomenon known as the reverse electron transfer (RET) (Hidalgo-Gutiérrez et al. 2021; Scialo et al. 2017). We suppose that impaired platelet mitochondrial metabolism in patients with post-COVID-19 syndrome can contribute to the reprogramming of mitochondrial OXPHOS toward glycolysis.

Viral infections induce production of reactive oxygen species, which can contribute to the alterations of mitochondrial bioenergetics. Different viruses are able to modulate antioxidant enzymes (Singh et al. 2021; Hidalgo-Gutiérrez et al. 2021). In our patients, the endogenous CoQ_10_ levels and TBARS in plasma of patients with post-COVID-19 syndrome were similar to control data, probably as a result of therapy with oxygen and drugs with antioxidant properties before starting MR.

High-altitude of the mountain spa environment improved mitochondrial fitness as could be seen from improved CI-linked OXPHOS and ET capacity of platelet mitochondria of patients with post-COVID-19 syndrome (Fig. 3, Fig. S3E – S3G). In MR2 group, platelet mitochondrial CI-linked LEAK respiration (*L*) was significantly increased vs MR1 (Fig. 3, Fig. S3B). The parameter *P-L* control efficiency (Gnaiger 2020) calculated from ADP-stimulated and LEAK reaspiration as (*P-L*)/*P* was slightly lower in the MR1 group vs controls, and after MR declined by 9.5% vs MR1 (*p* = 0.055) (Fig. 3, Fig. S3H). This parameter with values from 0 to 1 is a measure of coupling control efficiency. The mechanisms leading to decreased *P-L* control efficiency after MR in patients with post-COVID-19 syndrome could be a matter of further research. It could be speculated that increased physical activity in MR could induce oxidative stress mediating higher proton conductance of inner mitochondrial membrane at high proton motive force (at LEAK state), preventing this way increased ROS production by platelet mitochondria. An increase in LEAK respiration and a decrease in *P-L* control efficiency was found in platelets of ultramarathon runners after the race, reflecting increased proton leakage across the inner mitochondrial membrane (Hoppel et al. 2021). The increased CS activity in platelets of patients with post-COVID-19 syndrome may indicate increased density of mitochondria as a compensation for their decreased function.

## Conclusions

Comprehensive strategy for virus pandemic has to be based on medical evidence, on effective vaccines to decrease mortality, to improve economic growth and socioeconomic system. Spa rehabilitation in high-altitude environment contributes to the acceleration of patients’ health and to the reduction of socioeconomic problems. Our pilot findings contribute to the understanding of the role of mitochondria in the pathogenesis of COVID-19. Mountain spa rehabilitation can be recommended for the acceleration of recovery of patients with post-COVID-19 syndrome.

Limitations of these pilot results include relatively short time of mountain spa rehabilitation (16–18 days) paid by the insurance company and number of patients with post-COVID-19 syndrome (*n* = 14).

## Supplementary Information

ESM 1 (DOCX 110 kb)

## Data Availability

The supporting data are available from the authors upon request.
