# Supplementary material for: Mountain spa rehabilitation improved health of patients with post-COVID-19 syndrome: pilot study
Source: Environ Sci Pollut Res Int. 2022 Sep 23;30(6):14200–11. doi: 10.1007/s11356-022-22949-2 (PMC9510276; doi:10.1007/s11356-022-22949-2)
Supplement: Supplementary file 1 — (DOCX 110 kb) [file 11356_2022_22949_MOESM1_ESM.docx]

Gvozdjáková et al., ESPR 2022

**Supplementary material**


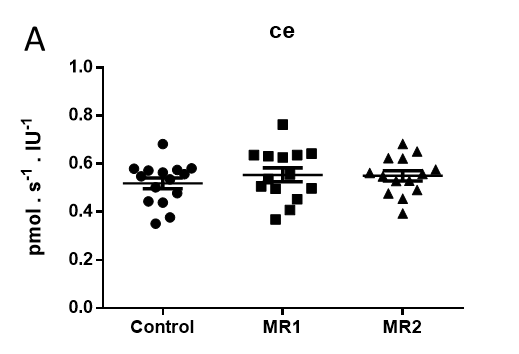

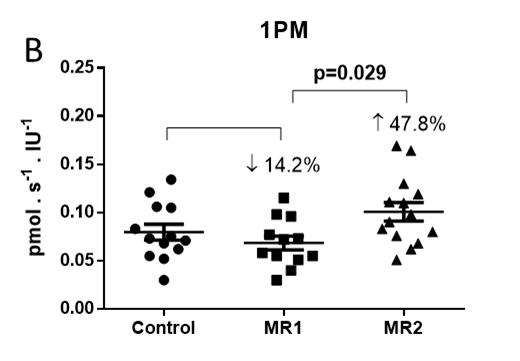


**
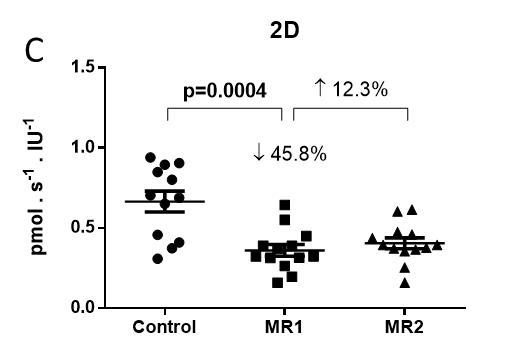

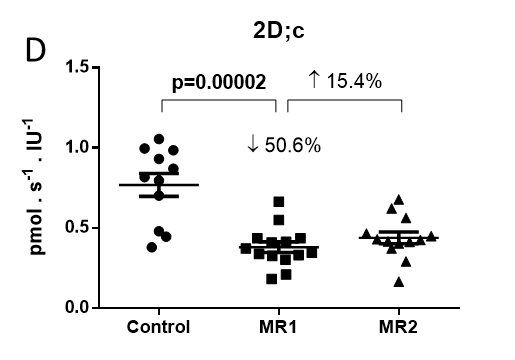
**


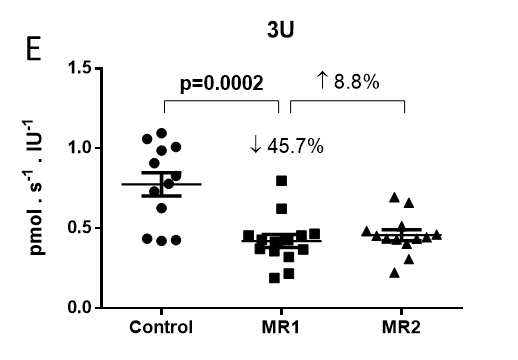

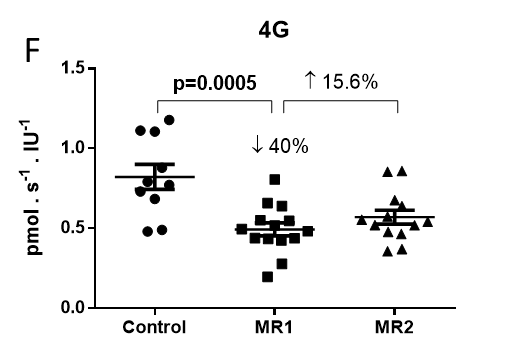


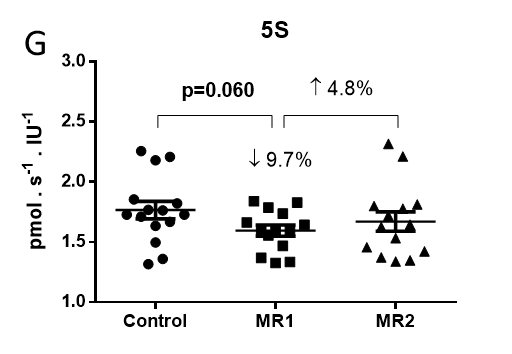

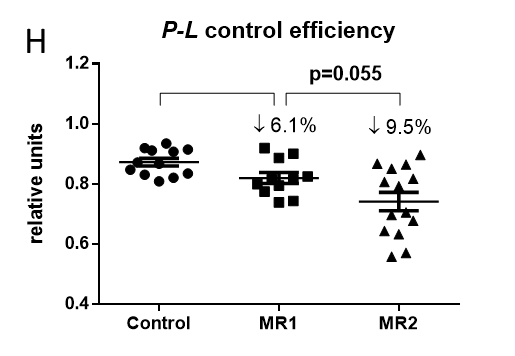


**Fig. S3** Effect of MR on platelet mitochondrial bioenergetics in patients with post-COVID-

19 syndrome

*Legend***: A)** ce: ROUTINE respiration of intact platelets; **B)** 1PM: Complex I-linked LEAK (State 4) respiration with substrates (pyruvate + malate); **C)** 2D: Complex I-linked OXPHOS (State 3) respiration capacity associated with CI-linked ATP production; **D)** 2D;c: The OXPHOS capacity after cytochrome c addition; **E)** 3U: The respiration after uncoupler FCCP titration represents CI-linked electron transfer (ET) capacity with substrates pyruvate+malate; **F)** 4G: ET capacity with substrates pyruvate+malate+glutamate; **G)** 5S: CI&II-linked ET capacity with substrates pyruvate + malate + glutamate + succinate, (Doerrier et al. 2016, Gvozdjáková et al. 2019). The respiratory rates are marked according the steps in the SUIT protocol 1 (see Fig 3). **H)** Effect of MR on *P-L* control efficiency in platelet mitochondria of patients with post-COVID-19 syndrome. Control – the control group; MR1 – patients before mountain spa rehabilitation; MR2 – patients after mountain spa rehabilitation.
